# The BMI Paradox and Robotic Assisted Partial Nephrectomy

**DOI:** 10.3389/fsurg.2019.00074

**Published:** 2020-01-09

**Authors:** Ohad Kott, Borivoj Golijanin, Jorge F. Pereira, Alison Chambers, Alison Knasin, Christopher Tucci, Dragan Golijanin

**Affiliations:** ^1^Division of Urology, Minimally Invasive Urology Institute, The Miriam Hospital, Providence, RI, United States; ^2^Warren Alpert Medical School of Brown University, Providence, RI, United States; ^3^Department of Pathology and Laboratory Medicine, Warren Alpert Medical School of Brown University, Providence, RI, United States; ^4^Division of Urology, Mount Sinai Medical Center, Columbia University, Miami Beach, FL, United States; ^5^Department of Medicine, Warren Alpert Medical School of Brown University, Providence, RI, United States; ^6^Department of Chemistry, Boston University, Boston, MA, United States

**Keywords:** partial nephrectomy, minimally invasive, obesity paradox, BMI, complications

## Abstract

**Introduction:** Partial nephrectomy (PN), has become the gold standard for the surgical management of small renal masses, due to excellent oncologic control with concomitant preservation of nephron units. However, data regarding the association of obesity with perioperative outcomes following PN are mixed. Therefore, the association between obesity (using BMI) and post-operative complications (POC) rate following Robotic assisted laparoscopic PN (RPNx) was tested.

**Methods:** Two hundred and fifty-one adult patients who underwent RPNx from 1/2011 to 5/2017 at a single institution, with at least 90 days follow-up were identified and included. No patients were excluded. Electronic medical records were reviewed to record all POC within 90 days of surgery. A piecewise generalized linear model for binary outcomes (logistic) was used to model the proportion of subjects with POC by their BMI. The slope of the line is adjusted to a BMI of 30 Kg/m^2^.

**Results:** BMI is significantly associated with POC rate. POC rate decreased with increasing BMI below the inflection point of 30 Kg/m^2^ (0.848[0.756, 0.952]) (OR [95% CI], *p* = 0.005). POC rate was found to increase with increasing BMI above the BMI inflection of 30 Kg/m^2^ (1.102 [1.027, 1.182], *p* = 0.0071).

**Conclusions:** In this cohort study, BMI showed an association with PC. It may be important to take BMI into account in surgical and clinical management considerations of RPNx, since higher rates of POC are associated with patients who are underweight, morbidly obese, and even with normal BMI. Further research is required on larger cohorts of RPNx patients to provide better description of this phenomenon and elucidate the role of BMI in development of POC.

## Introduction

Partial nephrectomy (PN), has become the gold standard for the surgical management of small renal masses, due to excellent oncologic outcomes with concomitant preservation of nephron units (1–3). Furthermore, when compared to open partial nephrectomy (OPN) minimally invasive partial nephrectomy (MIPN) has been associated with improved perioperative outcomes (4–8), including fewer post-operative complications (POC), shorter operative time, and a decreased length of stay (9, 10). Robotic assisted laparoscopic partial nephrectomy (RPNx) has become the most common method of MIPN due to its additional benefits over laparoscopic partial nephrectomy (LPN) such as shorter warm ischemia time (WIT) (11), lower rates of positive surgical margins, lower complication rates and enhanced nephron sparing (12). In addition to improving surgical techniques, it is also important to identify modifiable patient factors that may improve postoperative outcomes such as body mass index (BMI).

Elevated BMI is associated with several comorbidities that are associated with poor surgical outcomes (13), and has been shown to be an independent predictor of increased perioperative morbidity (14–17). Specifically, increased BMI is associated with a higher rates of surgical site infections, medication dosage errors, difficult ventilation, positioning related injuries, and postoperative mortality rates (18–21). While urologic data has focused on the effect of obesity on perioperative morbidity, literature from general surgery demonstrates a paradoxical relation between BMI and surgical outcomes (22–24). Recent studies have demonstrated a BMI paradox whereby lower mortality rates were noted among the overweight and the mildly obese patients, while increased mortality rates were seen in the underweight and extremely obese populations (23, 24). Evaluation of the BMI paradox in urologic surgery is lacking, and as such we seek to test the association between BMI and POC (POC) following RPNx.

## Methods

After institutional review board approval, we conducted a retrospective chart review and identified 251 adult patients who consecutively underwent RPNx for the treatment of a renal mass at our medical center between January 2011 and May 2017. All procedures were performed using the da Vinci surgical system (Intuitive Surgical, Sunnyvale, CA) by a single surgeon experienced in robot-assisted laparoscopic urological surgery. All procedures were done using similar surgical techniques as previously described (25).

Electronic medical records were reviewed to identify all POC within 90 days of surgery, graded according to the Clavien-Dindo system (26). Original analysis analyzed major and minor complications separately; however, only six major complications were found in our patient population so major and minor complications were grouped together as POC for all subsequent analysis. Patients' baseline characteristics recorded included: age, gender, smoking history, BMI, anticoagulants use, and medical comorbidities (diabetes, Hypertension, cardiac arrhythmia, coronary artery disease, COPD, GERD). Tumor characteristics recorded included: tumor size and RENAL nephrometry scores (27). Perioperative variables recorded included: operative time, estimated blood loss (EBL), abdominal insufflation volume, post-operative ambulation time, and length of stay (LOS). Patient information was collected and managed using REDCap electronic data capture tools hosted at Lifespan. No patients were excluded from this cohort or the data analysis.

### Statistical Methods

Medians and interquartile range (IQR) were used to report continuous variables. Frequencies and proportions were used to report categorical variables.

A generalized linear model for binary outcomes (logistic, *proc glimmix*) was used to model the proportion of subjects with POC by their BMI. A piecewise approach allowed the slope of the line to adjust at the BMI of 30. BMI 30 was selected as an adjustment point as it is accepted by the World Health Organization as lower range of obesity. From the model the odds ratios (slopes) were estimated below 30 BMI and above 30 BMI. Additionally, the probabilities of POC were estimated at BMIs of 20, 30, and 40 Kg/m^2^.

A receiver-operator characteristic (ROC) curve was generated from the multivariate model and AUC was used to assess ability of BMI to discriminate between presence and absence of POC. Optimal cutoffs (Sensitivity-specificity) were also determined from the model both below and above BMI of 30. The model was also assessed with an interaction term (multi-variate analysis) to understand the influence of confounders (operation time, diabetes, hypertension, and age) on the relationship between BMI and POC. Statistical analysis and hypothesis testing were performed using SAS (version 9.2; SAS Institute Inc., Cary, NC).

## Results

**Figure 1:**
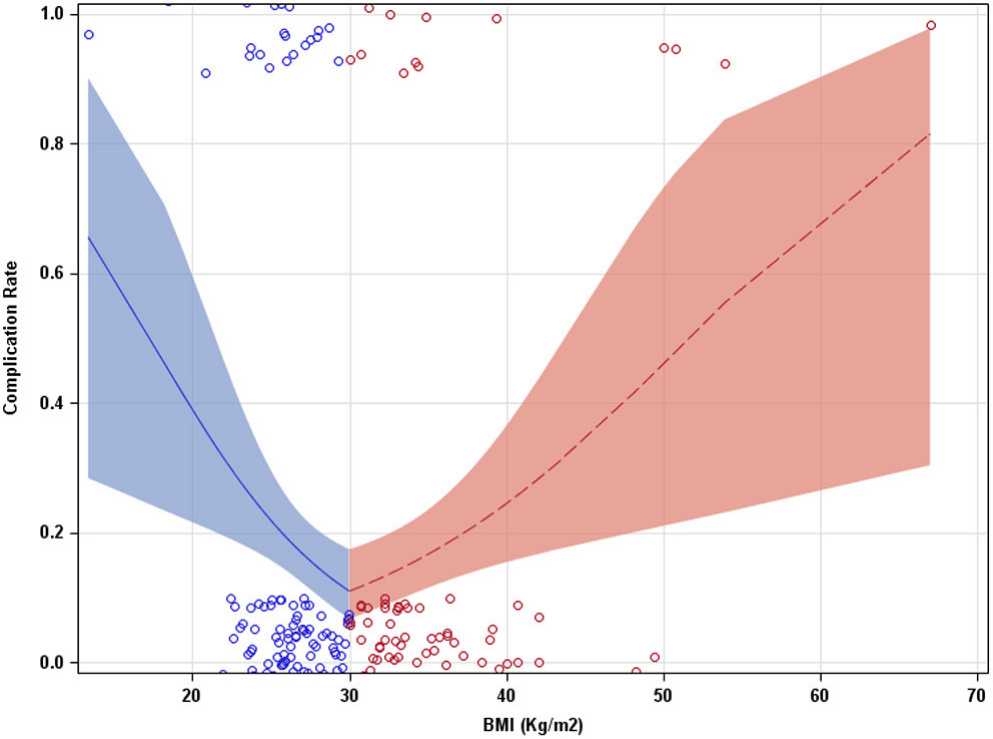
Complication rate as a function of BMI (Kg/m^2^). A piecewise approach allowed the slope of the model to adjust at a BMI of 30. Bands indicate 95% confidence intervals.

Our study cohort included 251 patients. Patient baseline characteristics are reported in Tables 1, 2. With the multivariate model, the odds of having POC were found to be significantly below 1 for BMIs under 30 Kg/m^2^ (0.85[0.76, 0.95]) (odds ratio [95% CI], *p* = 0.005). The odds were found to be significantly above 1 over the BMI inflection of 30 Kg/m^2^ (01.10[1.03, 1.18], *p* = 0.007) (Figure 1). Odds below 1 indicate a decrease in probability of POC, while odds above 1 indicate an increase in probability of POC. Probabilities of a POC at 20, 30, and 40 BMI were 0.39[0.22, 0.60], 0.11[0.07, 0.18], and 0.24[0.15, 0.36], respectively.

**Table 1:** Patient demographics and characteristics by BMI group.

**Variable**		**All patients**	**BMI < 30**	**BMI ≥ 30**
Number of patients (*n*)		251 (100%)	144 (57.37%)	107 (42.63%)
Age (years)		60.75	61.5 (54, 71)	60 (53, 68)
Operation time (minutes, IQR Q1, Q3)		459.5	215 (182, 242)	244.5 (202, 277)
Estimated blood loss (mL, IQR Q1, Q3)		100	100 (50, 200)	100 (75, 200)
Insufflation volume (L, IQR Q1, Q3)		7.5	3.6 (3, 4.3)	3.9 (3.1, 5)
Length of stay (days, IQR Q1, Q3)		6	3 (2, 4)	3 (2, 4)
Average tumor size (cm, IQR Q1, Q3)		5.1	2.4 (1.9, 3.5)	2.7 (1.9, 4)
Sex (Male %)		131 (52.19%)	77 (30.68%)	54 (21.51%)
Current smoker (*n*, %)		37 (14.7%)	23 (10.27%)	14 (6.25%)
Comorbidities	Diabetes	50 (19.9)	23 (9.16%)	27 (10.76%)
	Hypertension	159 (63.35%)	84 (33.47%)	75 (29.88%)
	Cardiac arrhythmia	11 (4.4%)	6 (2.39%)	5 (1.99%)
	CAD	13 (5.2%)	4 (1.59%)	9 (3.59%)
	COPD	12 (4.8%)	6 (2.39%)	6 (2.39%)
	GERD	59 (23.5%)	31 (12.35%)	28 (11.16%)
Nephrometry score	4–6	87 (34.7%)	50 (19.92%)	37 (14.74%)
	7–9	75 (29.8%)	42 (16.73%)	33 (13.15%)
	10–12	22 (8.8%)	12 (4.78%)	10 (3.98%)
	Missing	67 (26.7%)	40 (15.94%)	27 (10.76%)
Ambulation time	POD 0	29 (11.6%)	21 (8.37%)	8 (3.19%)
	POD 1	115 (45.8%)	72 (28.69%)	43 (17.13%)
	POD 2 and after	59 (23.5%)	25 (9.96%)	34 (13.55%)
	Missing	48 (19.1%)	26 (10.36%)	22 (8.76%)
Major complications (Clavien-Dindo > 2)		6 (2.39%)	5 (1.99%)	1 (0.4%)
All complications (Clavien-Dindo ≤ 2)		45 (17.93%)	26 (10.36%)	19 (7.57%)

**Table 2:** Fixed effects for multivariate models with interaction terms.

**Effect**	***P*-value**	**Effect**	***P*-value**	**Effect**	***P*-value**	**Effect**	***P*-value**
BMI	0.0761	BMI	0.0181	BMI	0.009	BMI	0.0031
BMI > 30	0.0178	BMI > 30	0.0136	BMI > 30	0.0029	BMI > 30	0.0091
Age	0.4085	Diabetes	0.5177	HTN	0.5904	Op time	0.6862
BMI*Age	0.4983	BMI*Diabetes	0.6799	BMI*HTN	0.6193	BMI*Op time	0.8535
BMI > 30*Age	0.5807	BMI > 30*Diabetes	0.981	BMI > 30*HTN	0.9914	BMI > 30*Op time	0.9738

**Figure 2:**
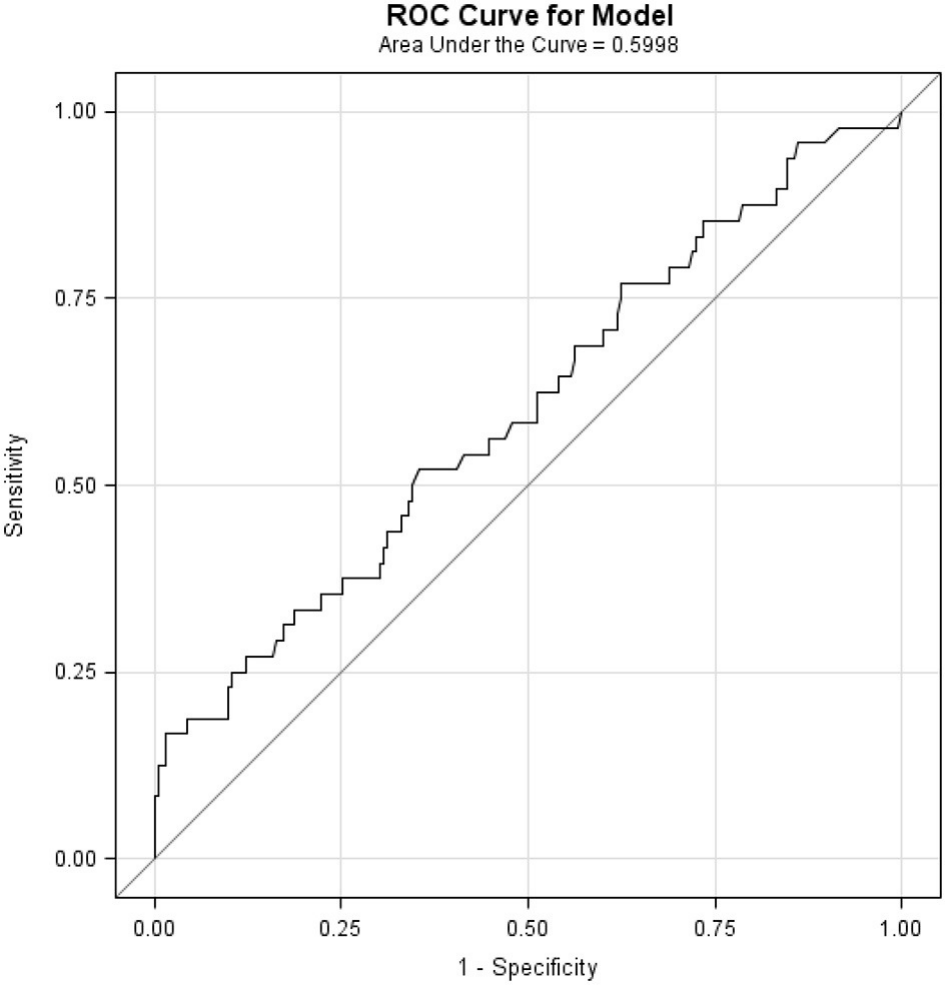
Receiver-Operator Characteristic (ROC) Curve. Area under the curve (AUC) of 0.5998 shows the ability of BMI to discriminate between presence and absence of POC. Perfect accuracy is defined as an AUC of 1.0.

ROC analysis showed an AUC of 0.5998 (accuracy of 59.98% for BMI to discriminate between presence and absence of POC) (Figure 2). Perfect accuracy is defined as an AUC of 1. The optimal BMI cut offs (determined using sensitivity-specificity) for the lower part and upper part of the curve were 26.7 and 35.7 Kg/m^2^, respectively. When the model was also assessed with interaction terms to test the separate influence of confounders (operation time, diabetes, hypertension, and age) on the relationship between BMI and PC. Confounders where not found to significantly contribute to the model (all *p* ≥ 0.4983, Table 2), while BMI was still found to be significantly related to POC in all models (Table 2).

## Discussion

In this retrospective single institution study, we have shown that BMI was associated with POC rate in patients undergoing RPNx. The rate of POC (odds) was found to be lower with increasing BMI up until the BMI inflection point (30 Kg/m^2^). Above the BMI of 30 the POC rate (odds) was found to be higher. This suggests that underweight and morbidly obese patients have the greatest risk of developing POC. This may indicate a paradoxically lower risk for POC after RPNx with overweight and mildly obese patients compared with patients at a normal weight. Our result are consistent with several studies examining large patient populations undergoing non-bariatric surgical procedures that demonstrated a paradoxical association between BMI and POC (23, 28, 29). These previous studies found the association function to have a function curve with a nadir of lowest complications rate at BMI between 25 and 35 Kg/m^2^. This is similar to our study where we found, the estimated probabilities (complication rate) at a BMI of 20 or 40 had a higher rate of POC than those with a BMI of 30. Additionally, our study saw cutoff values for BMI at 26.7 and 35.7 Kg/m^2^, with patients in the range between these two values having the lowest probability of PC. Although higher rates of POC are observed in underweight and obese patients, these results do not prove causality and interdependence between BMI and PC.

Our study and the aforementioned general surgery literature, stands in contrast to studies that found no association between BMI and surgical outcomes (30) or described a direct linear relationship showing that obesity is associated with increased risk of morbidity and mortality including cancer (31, 32) and higher renal mass complexity (33). One such study specifically examining LPN concluded that high BMI was associated with increased risk of major complications in patients who underwent LPN (34). Others found BMI to be a predictor of poor surgical outcomes in open partial nephrectomies but not in LPN (35). This may be explained by the excess of perinephric fat that requires longer dissection during PN. Perinephric fat was found to be significantly correlated with operative time in renal procedures while BMI had no correlation with operative time (36). However, other studies found no association between obesity (either measured by visceral and perinephric fat or by BMI) and surgical outcomes in RPNx (37, 38). When looking at a large study population, such as the NSQIP data base, there was no association between BMI and POC after OPN and MIPN (39). This study concluded that obese patients undergoing MIPN had lower POC rate than those undergoing OPN. However, these studies may have overlooked a more complex relationship between BMI and PC, as they did not allow for changes in POC rate with increasing BMI. Not allowing for POC rate changes with increasing BMI could underestimate probabilities of low BMI individuals and overestimate probabilities of moderate BMI individuals.

The accuracy of BMI in predicting POC was 59.98%, which indicates that there may be other factors that could help explain the remaining uncertainty in the prediction of PC. The confounders of operation time, patient age, anticoagulant use comorbidities (e.g., diabetes, hypertension) were evaluated for their ability to help define the remaining uncertainty in the multivariate model. Each confounder term was added to the model as an interaction term to test to see if they provided an alternative prediction of POC or if they modified BMI's relationship to PC. The influence of the confounders did not significantly contribute to the model (all interaction terms *p* ≥ 0.4983). This suggests that while these confounders may have an independent relationship with POC, they do not appreciably modify the association of BMI with POC and do not help explain the remaining uncertainty in the model.

Additionally, the number of POC may be too small to detect a more subtle effect of these modifiers on the outcome. Alternatively, the adverse effects of low BMI may be mediated by metabolic and inflammatory responses that occur after major surgical procedures like partial nephrectomy. It was previously suggested (23) that patients who are overweight or mildly obese may experience a protective effect against the inflammatory response and increased metabolic demands associated with the physical insult of surgery due to larger nutritional reserves and a more efficient metabolic state than underweight patients and even normal weight patients. If this hypothesis is correct, mildly obese patients may have better response to the metabolic and inflammatory stress of surgery, engage faster tissue repair even in the setting of low caloric and protein intake.

The change in inflammatory response amongst overweight and mildly obese patients is thought to be modulated via the immune system. One theory proposes that moderate amounts of adipose tissue may protect against inflammation via the secretion adipokines such as adiponectin, IL-1, IL-6, and soluble TNF-α receptors which work to neutralize endotoxins (40). As such, obese patients may normally live in constant state of mild inflammation, from metabolic reasons, and are adapted to it. Therefore, after surgery they may quickly respond to surgical injury, stress, and inflammation and engage in immune response and tissue repair.

This study is not without limitations. To limit possible confounding factors, the study population was limited to surgical patients operated by a single, highly specialized surgeon in a tertiary referral center. As such, there may be some level of selection bias in the type of patients that present to the medical center. Our findings may not reflect other surgeons in other clinical settings. Furthermore, while this study's cohort size is larger than several recently published studies evaluating partial nephrectomy outcomes (33–36), this study size is still too small to allow for a full detailed analysis of the complex relationship between BMI, tumor characteristics, and POC.

In conclusion, patient BMI may be associated with increased risk of POC and should be considered during preoperative planning and patient counseling. Surgeons evaluating patients that are underweight, morbidly obese, and even with normal BMI should take that into consideration. Further research is required to further characterize and precisely quantify and delineate the exact effects of obesity over surgical outcomes of RPNx using a larger cohort of patients.

## Data Availability Statement

The datasets generated for this study are available on request to the corresponding author.

## Ethics Statement

The studies involving human participants were reviewed and approved by Lifespan—The Miriam Hospital IRB, Providence, RI. Ref# 214214 45CFR 46.110(5). Written informed consent for participation was not required for this study in accordance with the national legislation and the institutional requirements.

## Author Contributions

OK: project development, data collection and management, data analysis, manuscript writing, and editing. BG: data collection, data analysis, and manuscript editing. JP: project development, data analysis, and manuscript editing. AC: data analysis and manuscript editing. AK: project development, data collection, and manuscript editing. CT: project development and manuscript editing. DG: project conception, project development, and manuscript editing.

### Conflict of Interest

The authors declare that the research was conducted in the absence of any commercial or financial relationships that could be construed as a potential conflict of interest.

## Abbreviations

PN: Partial nephrectomy
BMI: Body mass index
POC: Post-operative complications
RPNx: Robotic assisted laparoscopic partial nephrectomy
OPN: Open partial nephrectomy
MIPN: Minimally invasive partial nephrectomy
LPN: Laparoscopic partial nephrectomy
WIT: Warm ischemia time
COPD: Chronic obstructive pulmonary disease
GERD: Gastroesophageal reflux disease
EBL: Estimated blood loss
LOS: Length of stay
IQR: Interquartile range
ROC: Receiver-operator characteristic
AUC: Area under the curve (in receiver operated probability curve)
CI: Confidence interval
NSQIP: National Surgical Quality Improvement Program (of American College of Surgeons).
